# Gallbladder polyps ultrasound: what the sonographer needs to know

**DOI:** 10.1007/s40477-021-00563-1

**Published:** 2021-02-06

**Authors:** G. Cocco, R. Basilico, A. Delli Pizzi, N. Cocco, A. Boccatonda, D. D’Ardes, S. Fabiani, N. Anzoletti, P. D’Alessandro, G. Vallone, F. Cipollone, C. Schiavone

**Affiliations:** 1 Unit of Ultrasound in Internal Medicine, Department of Medicine and Science of Aging, “G. D’Annunzio” University, Chieti, Italy; 2 Department of Neurosciences, Imaging and Clinical Sciences, “G. D’Annunzio” University, Chieti, Italy; 3 Departmental Faculty of Medicine and Surgery, Campus Bio-Medico University, Rome, Italy; 4 Department Life and Health “V. Tiberio, Università Degli Studi del Molise, Campobasso, Italy; 5 Clinica Medica Division and European Center of Excellence On Atherosclerosis, Hypertension and Dyslipidemia “SS. Annunziata” Hospital, Chieti, Italy

**Keywords:** Gallbladder, Ultrasound, Polyps

## Abstract

Gallbladder polyps are protuberances of the gallbladder wall projecting into the lumen. They are usually incidentally found during abdominal sonography or diagnosed on histopathology of a surgery specimen, with an estimated prevalence of up to 9.5% of patients. Gallbladder polyps are not mobile and do not demonstrate posterior acoustic shadowing; they may be sessile or pedunculated. Gallbladder polyps may be divided into pseudopolyps and true polyps. Pseudopolyps are benign and include cholesterolosis, cholesterinic polyps, inflammatory polyps, and localised adenomyomatosis. True gallbladder polyps can be benign or malignant. Benign polyps are most commonly adenomas, while malignant polyps are adenocarcinomas and metastases. There are also rare types of benign and malignant true gallbladder polyps, including mesenchymal tumours and lymphomas. Ultrasound is the first-choice imaging method for the diagnosis of gallbladder polyps, representing an indispensable tool for ensuring appropriate management. It enables limitation of secondary level investigations and avoidance of unnecessary cholecystectomies.

## Introduction

Gallbladder polyps are elevations of the gallbladder wall projecting into the lumen [1]. They are incidentally found during abdomen sonography or following cholecystectomy, with an estimated prevalence of up to 9.5% of patients [2]. Gallbladder polyps are not mobile and do not demonstrate posterior acoustic shadowing. They may be sessile or pedunculated. A systematic review by Elmasry et al. classified the gallbladder polyps into pseudopolyps and true polyps [3]. Pseudopolyps are benign and include cholesterolosis, cholesterinic polyps, inflammatory polyps, and localised adenomyomatosis. True gallbladder polyps can be benign or malignant. Benign polyps are most commonly adenomas, while malignant polyps are adenocarcinomas and metastases [2, 3]. Furthermore, there are rare types of benign and malignant true gallbladder polyps, such as mesenchymal tumours and lymphomas. In this pictorial review, we briefly recall the normal anatomy and function of the gallbladder, describing the normal ultrasonographic anatomy and pointing out a wide range of gallbladder wall pathological disorders. This review is based on the combined experience of our centres, with a thorough analysis of the literature from the past 18 years (2001–2019). A systematic search of the literature was performed in PubMed and included original studies and review articles dealing with sonographic descriptions of gallbladder polyps and related disorders. Case reports and case series were selected according to clinical relevance. We aim to provide sonographers with an ultrasound guide for gallbladder polyps to help interpret whether the polyp is at high or low risk of malignancy and to suggest patient management accordingly.

### Gallbladder anatomy

**Fig. 1:**
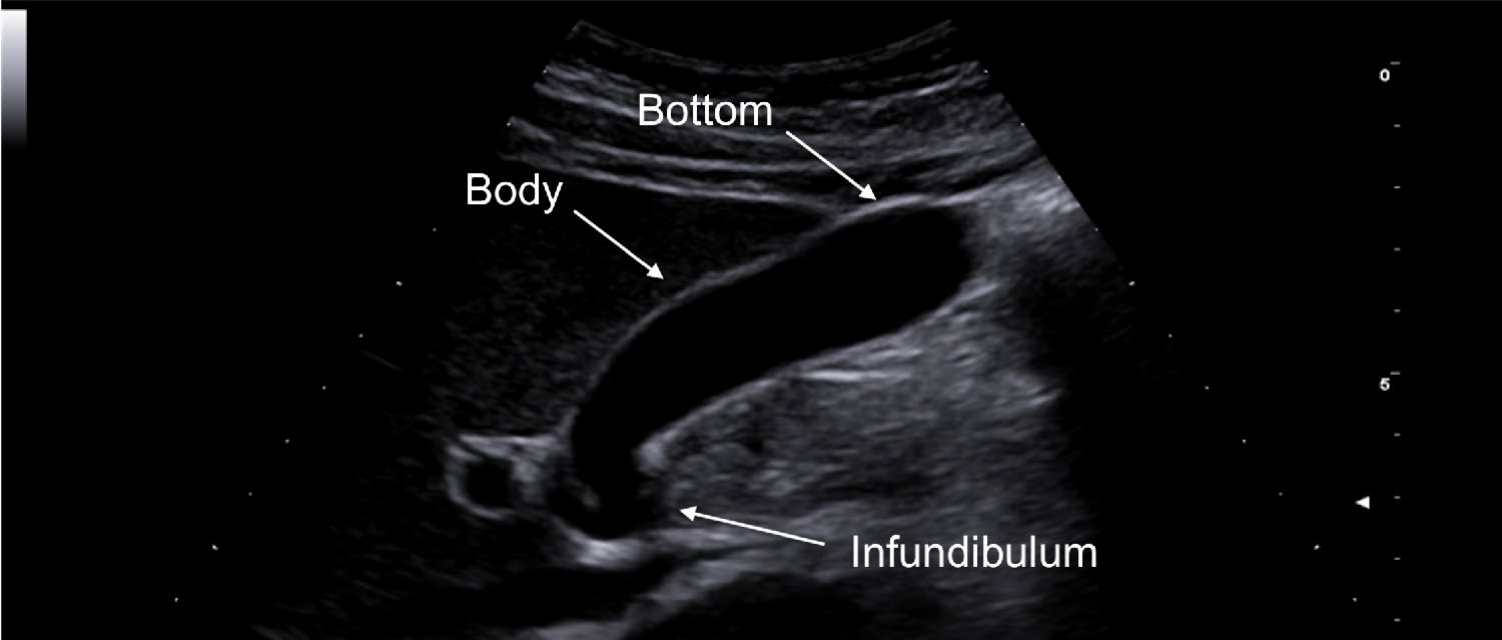
Sonography image depicts a representation of the gallbladder: infundibulum, body, and bottom (white arrows)

The gallbladder is a hollow, pyriform viscera that is ~ 7–10 cm long and ~ 2.5–3.5 cm wide with thin and regular walls. It is located in the gallbladder fossa between the IV and V segments of the liver, which is an area devoid of the visceral peritoneum [4]. The gallbladder is composed of three parts: infundibulum, body, and fundus, or bottom (Fig. 1). Its function is to store a volume of bile, normally 30–50 ml [5]. When food enters the small intestine, a hormone called cholecystokinin is released, signalling the gallbladder to contract and secrete bile into the small intestine through the common bile duct. The gallbladder acts as a reservoir that accumulates bile during fasting, pouring it into the initial tract of the small intestine after meals to aid the digestive process, especially by breaking up fats. According to several authors, the upper limit of normality for the gallbladder wall thickness is 4 mm [4]. The gallbladder parietal thickness may exceed this limit if the patient is not fasting because of the organ’s smooth muscle contraction or for certain pathological causes, including inflammatory, neoplastic, and systemic pathologies. These pathologies may be differentiated by means of a combined evaluation of clinical and imaging findings [6, 7].

**Fig. 2:**
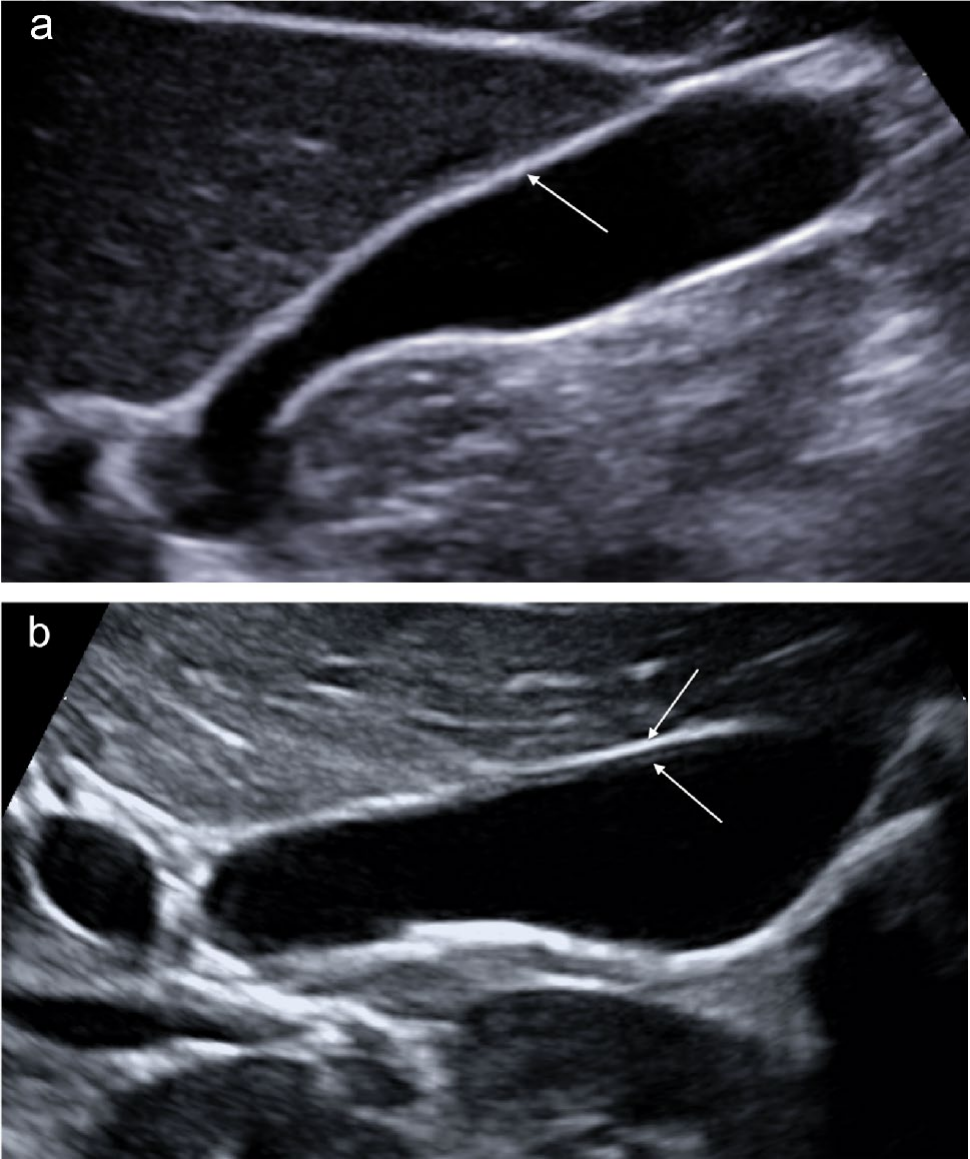
**a** Conventional transabdominal sonography image using a convex probe (1–5 MHz) shows a single layer (white arrow). **b** Conventional transabdominal sonography image using a convex probe (1–5 MHz) shows two layers of the gallbladder wall (white arrows)

**Fig. 3:**
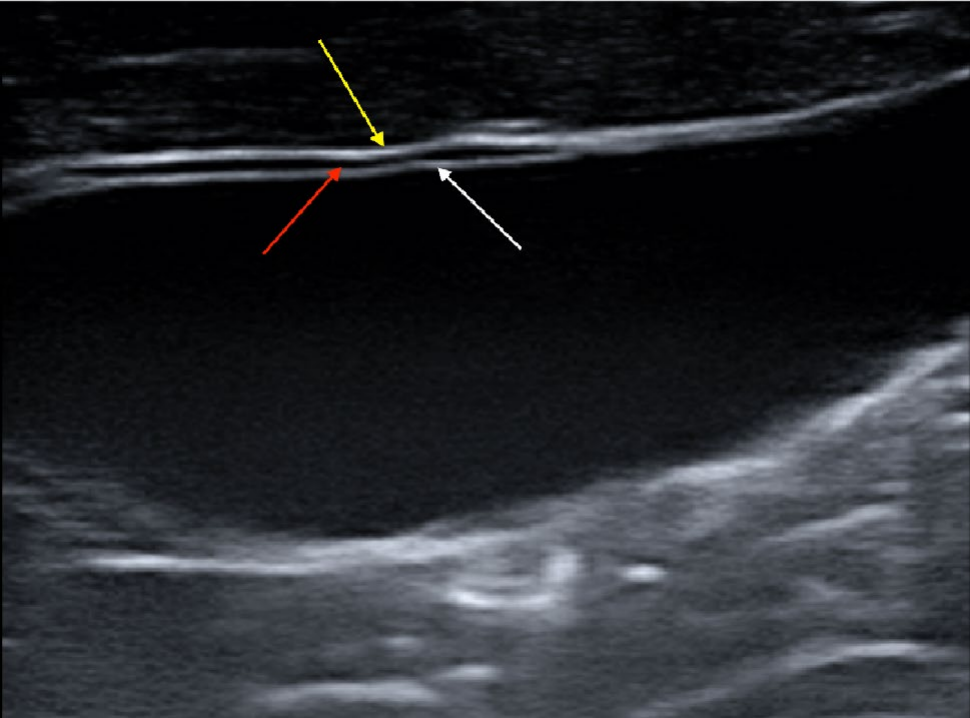
Linear probe using high-frequency transducers can better visualise the gallbladder wall layers. The image depicts three layers of the gallbladder wall including the innermost hyperechoic layer (white arrow), the middle thin hypoechoic layer (red arrow), and the outermost hyperechoic layer (yellow arrow)

Ultrasound provides an optimal representation of the whole gallbladder (infundibulum, body, and bottom). A conventional transabdominal sonography image using low-frequency transducers shows a single or two layers of gallbladder wall (Fig. 2a, b). High-resolution sonography (HRUS) using high-frequency transducers depicts three layers of gallbladder wall, including the innermost hyperechoic layer, the middle thin hypoechoic layer, and the outermost hyperechoic layer. The innermost layer corresponds to the mucosa and is linear, echogenic, and presents a regular surface; the middle layer corresponds to the muscular layer and is thin and slightly hypoechoic; and the outermost layer corresponds to the organ’s serosa and is linear, echogenic, and regular (Fig. 3) [4, 6].

### Gallbladder sonography: study technique

Gallbladder optimal sonography should be performed with the patient in a fasting state (about 6–8 h) using convex (2–8-MHz) and linear (3–13 MHz) transducers. The exam should be optimised to acquire appropriate B-mode images and carried out through systematic scanning with subcostal and intercostal approaches, and by longitudinal and cross-sectional views of the organ. Anatomy (shape, dimensions, wall thickness, regularity and texture patterns of the gallbladder walls) and contents should be evaluated.

**Fig. 4:**
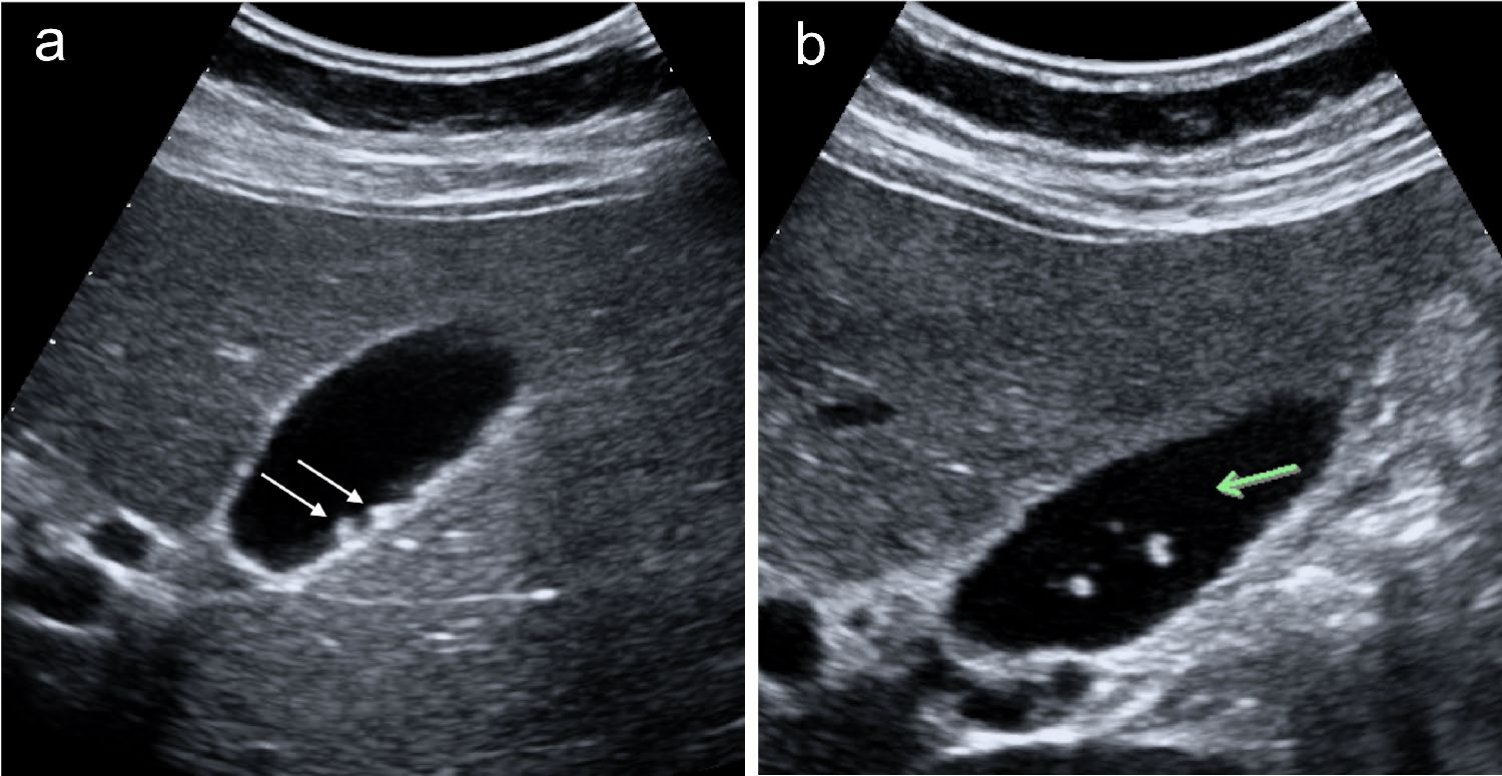
**a** The ultrasound images show fine deposits of biliary sludge that may simulate gallbladder wall polyps (white arrows). **b** It is useful to carry out a dynamic ultrasound of the gallbladder by changing the patient's decubitus (green arrow)

Fine deposits of biliary sludge may simulate gallbladder wall polyps (Fig. 4a). It is useful to carry out a dynamic ultrasound of the gallbladder by changing the patient’s position. The polyps will remain fixed as the biliary sludge comes off the wall (Fig. 4b). The gallbladder sonography should be integrated by a colour and power Doppler exam [8]. When the colour Doppler mode is used, the operator should set the machine with low PRF values and optimise the colour gain, to evaluate the flow of the small vessels of the gallbladder wall, characterised by low velocities. Currently, ultrasound machines are equipped with specific presets for slow vascular flows.

### New ultrasound techniques

High-resolution ultrasound (HRUS) is based on both low and high-frequency transducers in the evaluation of the gallbladder. Since high frequency is characterised by better resolution, HRUS can better evaluate the gallbladder wall layers and more accurately analyse the internal echo change of polyps in comparison to conventional transabdominal sonography. Although the data in the literature are still limited, HRUS seems to provide successful performance in evaluating gallbladder cancer and to differentiate gallbladder cancer from adenomyomatosis [9, 10].

3D-US is an emerging modality in the diagnostic evaluation of the gallbladder. A study performed on 80 patients with gallbladder polyps demonstrated that there was an agreement in the diagnosis in 89% of cases when 2D and 3D ultrasound was applied. However, 3D US showed worse diagnostic performance in detecting polyps of less than 4 mm [11].

Contrast-enhanced ultrasound (CEUS) is a promising tool to improve the diagnostic accuracy in the detection and evaluation of gallbladder diseases. Numata et al. [12] used galactose palmitic acid contrast injection to assess polyps by analysing the criteria of tumour enhancement and tortuous-type tumour vessels; this technique demonstrated 91% accuracy in diagnosing malignant lesions [12]. Other studies showed a limitation related to the size of the lesions because diagnostic accuracy was greater in polyps more than 10 mm in size [13].

### Cholesterolosis

Cholesterolosis is the most common pseudopolyp [2, 3, 14]. It is asymptomatic and can be isolated or associated with gallstones. Cholesterolosis is usually diagnosed incidentally during sonography. This condition results from abnormal deposits of lipids (triglycerides, cholesterol precursors, and cholesterol esters) into the gallbladder mucosa [2, 3].

**Fig. 5:**
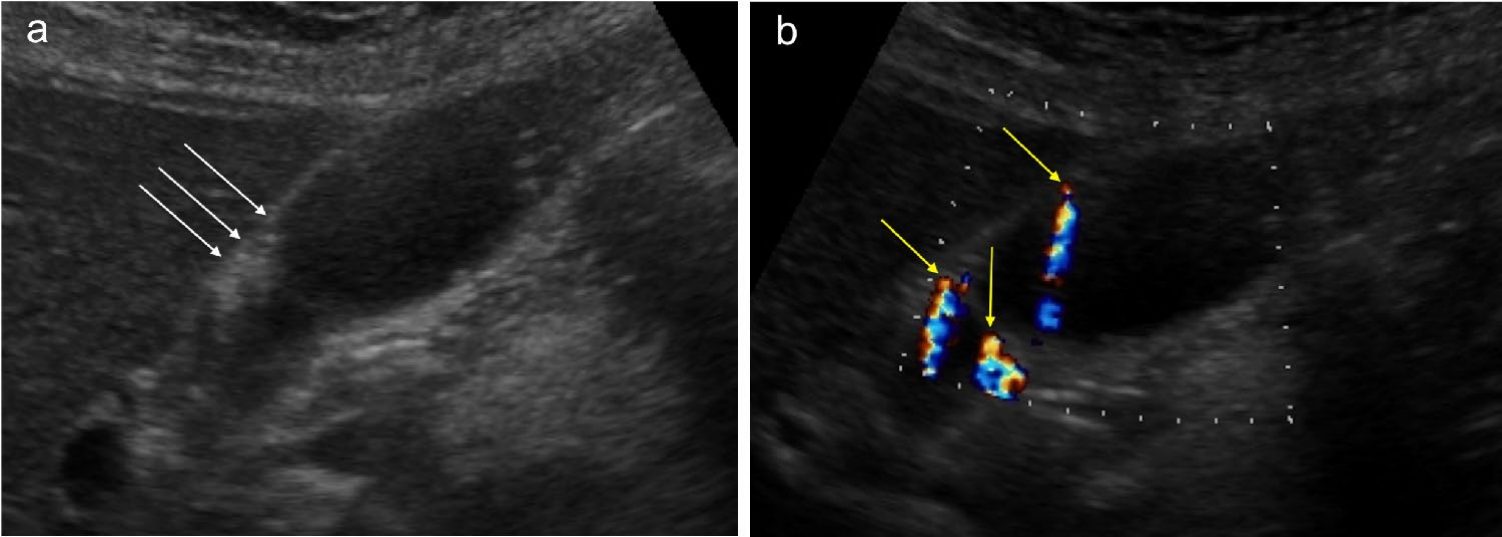
**a** Sonography image depicts parietal hyperechoic multiple foci on the gallbladder body and the infundibulum wall, generating comet-tail artifacts on B-mode (white arrows). **b** Sonography image depicts parietal hyperechoic multiple foci on the gallbladder body generating twinkling artifacts on the colour Doppler exam (yellow arrows)

During sonography, cholesterolosis appears as parietal hyperechoic single or multiple foci on the gallbladder wall, generating comet-tail artifacts on B-mode and twinkling artifacts on the colour Doppler exam. A comet-tail artifact is a form of reverberation (Fig. 5a). In this artifact, the two reflective interfaces and subsequential echoes are closely spaced. The result is an artifact caused by the principle of reverberation but with a triangular, tapered shape [15]. Twinkling artifacts on colour Doppler ultrasound are due to the interaction of the ultrasound beam with a rough acoustic interface composed of randomly disposed, strongly reflecting media such as cholesterol crystals or calcification (Fig. 5b) [16].

### Cholesterol and inflammatory polyps

**Fig. 6:**
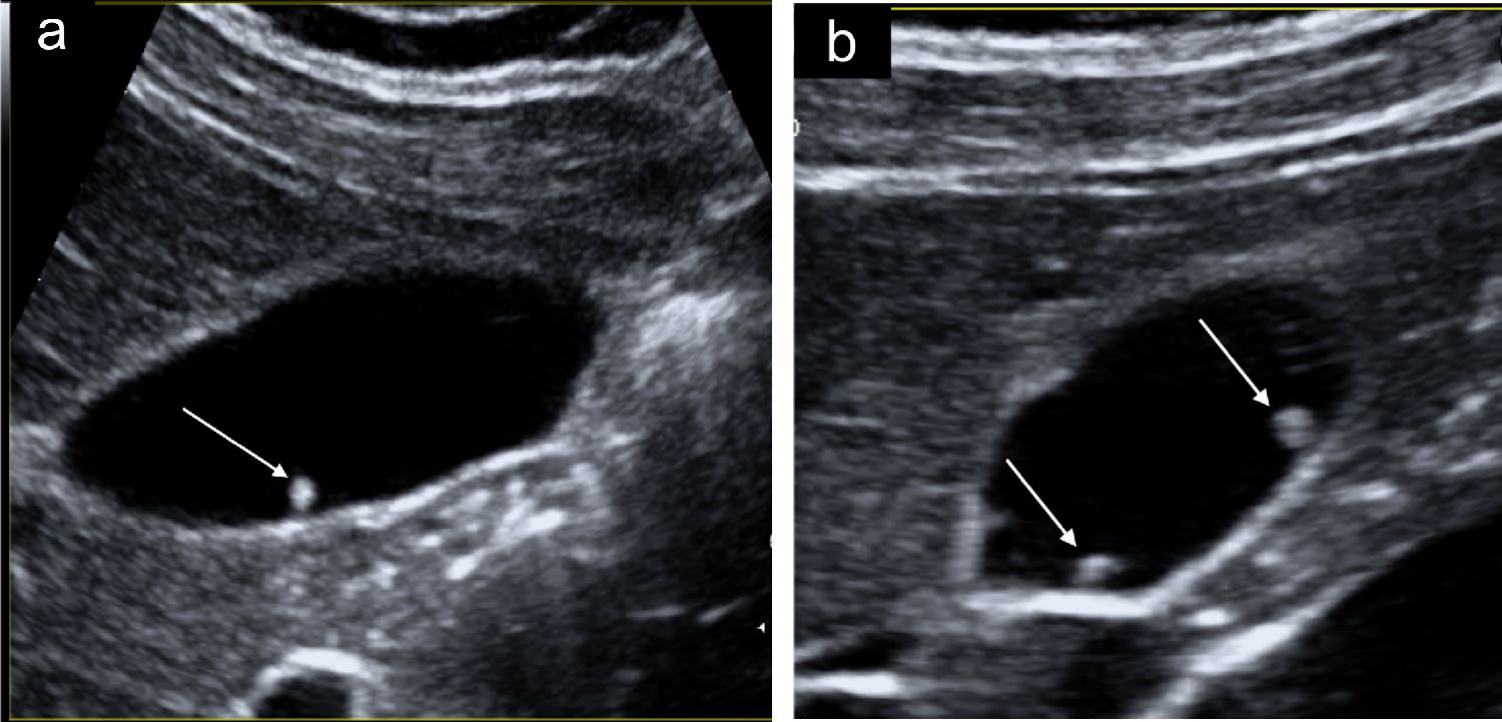
**a** The sonographic image shows solitary cholesterol polyps, homogeneous, pedunculated, without posterior acoustic shadowing, smaller than 1 cm, more hyperechoic than the liver parenchyma (white arrow). **b** The sonographic image shows two cholesterol polyps, homogeneous, pedunculated, without posterior acoustic shadowing, smaller than 1 cm, more hyperechoic than the liver parenchyma (white arrows)

Cholesterol polyps represent a polypoid variant of cholesterolosis, where the deposits give rise to solitary or multiple cholesterol polyps that are attached to the underlying mucosa with a fragile epithelial pedicle composed of lipid-filled macrophages. These polyps can break off, leading to complications similar to those caused by small gallstones. At sonography, cholesterol polyps typically appear as multiple lesions and are homogeneous, pedunculated, and smaller than 1 cm; polyps are generally more hyperechoic than the liver parenchyma (Fig. 6a, b) [2, 3, 17].

**Fig. 7:**
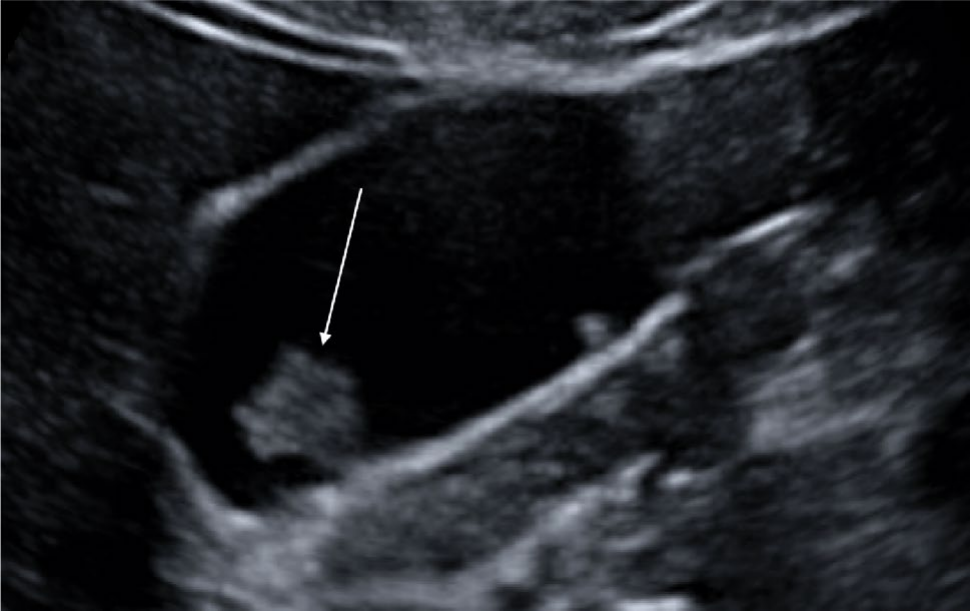
The sonographic image shows an inflammatory polyp (white arrow) that appears with a higher echogenicity than the liver parenchyma, homogeneous, pedunculated, and less than 10 mm in diameter

Inflammatory polyps are the least common type of pseudopolyp, accounting for about 10% of gallbladder polyps. They are local epithelial proliferations of inflammatory reactions with infiltration of inflammatory cells and are often associated with chronic cholecystitis. At sonography, inflammatory polyps are often characterised by higher echogenicity than liver parenchyma and are homogeneous, sessile or pedunculated (Fig. 7), hardly distinguishable from cholesterol polyps. Although inflammatory polyps larger than 1 cm have been described, they are usually less than 10 mm in diameter. Notably, large inflammatory polyps can be confused with true gallbladder polyps [2, 3].

Generally, the differential diagnosis between cholesterol and inflammatory polyps is histological and, for this reason, CEUS is not routinely used. These lesions usually appear homogeneous, slightly hyperechoic in the arterial phase and surrounded by normal tissue [18].

### Gallbladder adenomyomatosis

Gallbladder adenomyomatosis (GA) is a common benign pathology of the gallbladder wall and represents approximately 40% of benign gallbladder lesions [19]. It is asymptomatic and the diagnosis is infrequent during abdominal ultrasound exam. Gallbladder adenomyomatosis is characterised by excessive epithelial proliferation associated with hyperplasia of the muscularis propria, resulting in a thickening of the gallbladder wall. The excessive epithelial proliferation leads to epithelial infolding within the underlying muscular layer with subsequent formation of epithelium-lined diverticular pouches called Rokitansky–Aschoff sinuses (RAS) [19, 20]. The content of RAS consists of bile that may undergo progressive dehydration over time, leading to cholesterol crystal precipitation [19]. Cholesterol crystals may induce a chronic inflammatory reaction leading to the development of intramural dystrophic calcification. The serosa is never involved during GA [19].

Gallbladder adenomyomatosis is a benign alteration, but it can indirectly cause gallbladder carcinoma. This is due to the stiffness of the gallbladder wall, which causes the deposit of crystals, thus inducing cholelithiasis, a well-known risk factor for gallbladder carcinoma [21]. Sonography can be very useful in monitoring GA, especially without the risk of cholelithiasis or unnecessary cholecystectomies. Gallbladder adenomyomatosis may increase in size over time and this change alone must not be considered an index of malignancy. In any case, the surgical option may be considered in patients with segmental-type GA, given its higher association with gallbladder cancer, and in patients with diffuse GA, given the possible difficulties in identifying neoplastic foci within the wall thickening [19–22].

**Fig. 8:**
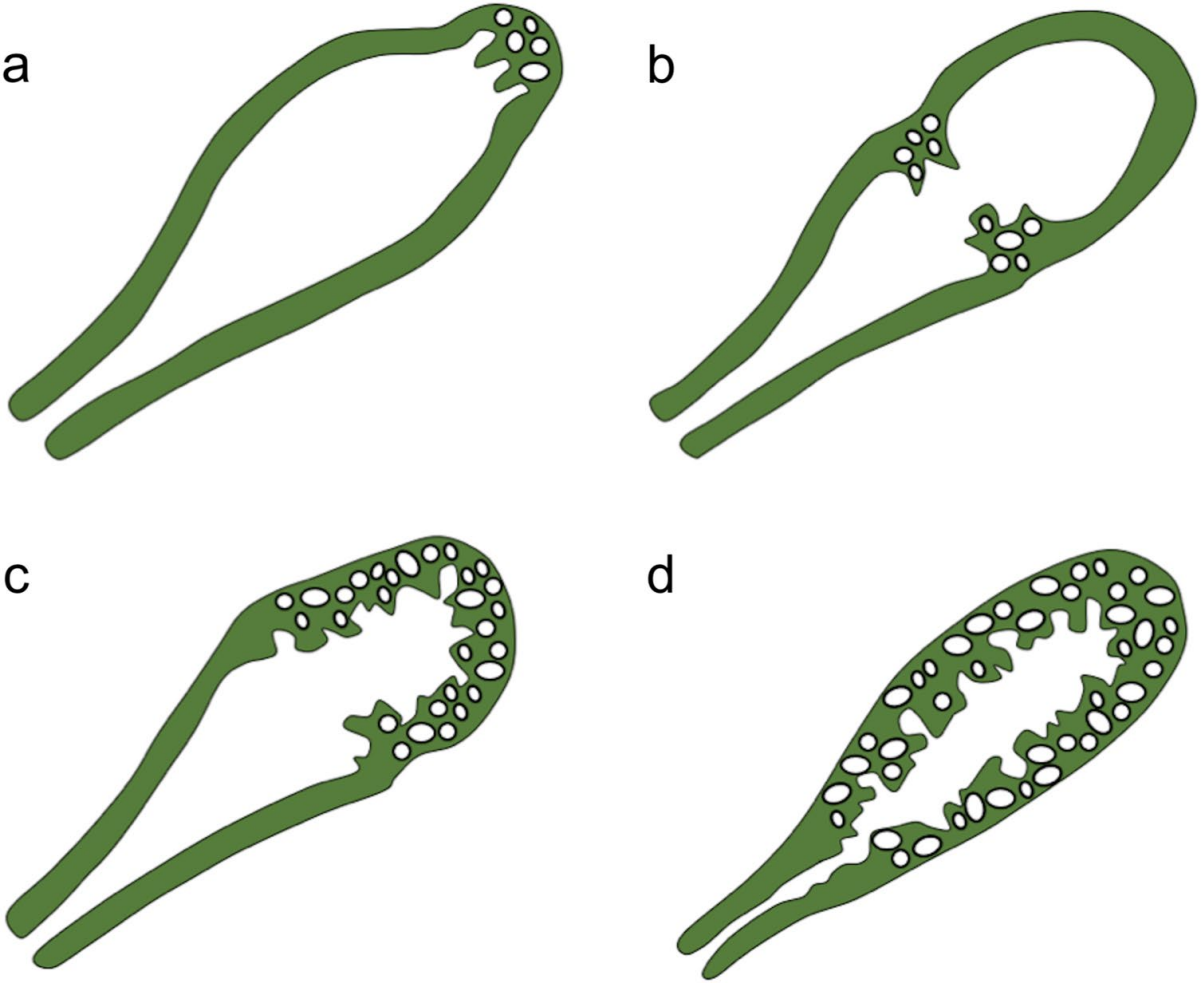
Gallbladder adenomyomatosis patterns: **a** localised, **b** annular, **c** segmental, and **d** diffuse

The preferential imaging modality for diagnosing GA is ultrasound. At sonography, the RAS typically appear hypoanechoic, usually observed along with hyperechoic cholesterol crystals or calcifications generating comet-tail reverberation artifacts or acoustic shadowing on B-mode images and twinkling artifacts on colour Doppler images [19–23]. Adenomyomatosis may involve the gallbladder according to four main patterns: localised, segmental, annular, and diffuse (Fig. 8) [19, 24, 25]. Sonographic detection and characterisation of GA demonstrate accuracy values that range from 91.5 to 94.8% in differentiating GA from early-stage gallbladder cancer [26].

**Fig. 9:**
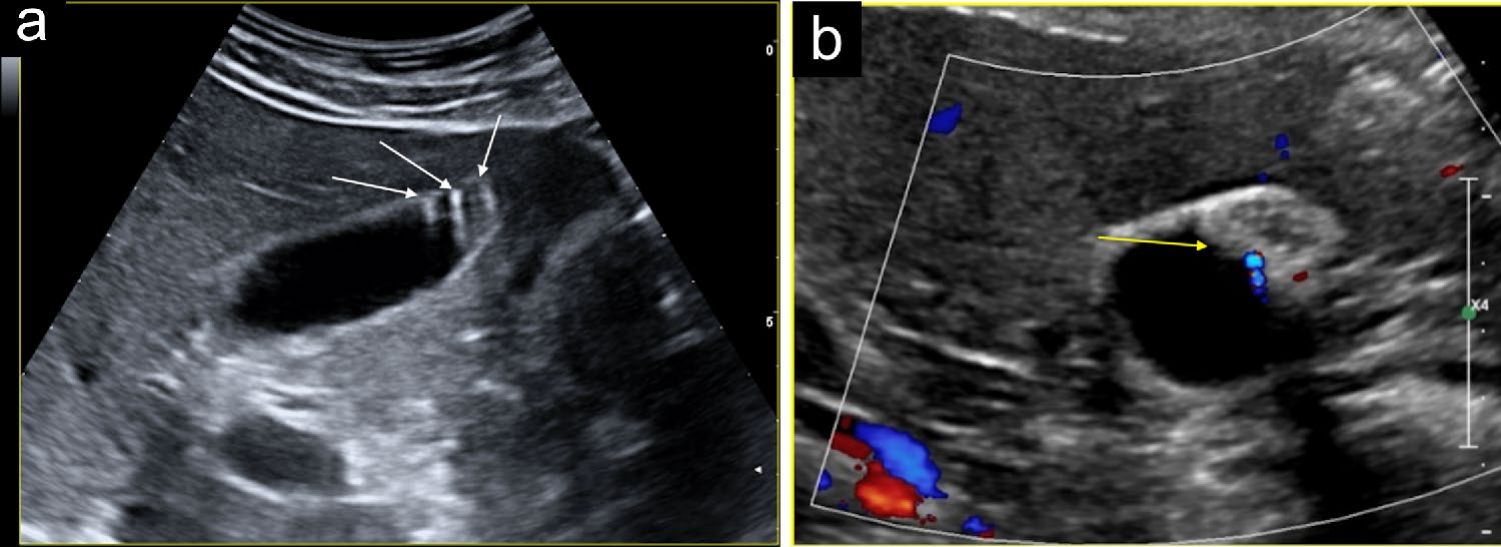
**a** The sonographic images show the localised type of GA as a focal thickening localised in the fundus. The thickening projects into the lumen and appears hypoanechoic with hyperechoic cholesterol crystals generating comet-tail reverberation artifacts (white arrows). **b** The sonographic images show the localised type of GA as a focal thickening localised in the fundus. The thickening projects into the lumen and appears hypoanechoic with hyperechoic cholesterol crystals generating twinkling artifacts on the colour Doppler exam (yellow arrow)

The localised type of GA is the most common pattern, where the focal thickening is usually in the fundus. The thickening projects into the lumen in a similar way to a polyp. The RAS typically appear hypoanechoic or with hyperechoic cholesterol crystals or calcifications and twinkling artifacts (Fig. 9a, b). Sometimes localised GA can simulate a polyp lesion and in these cases CEUS can increase the degree of visualisation of (RAS) as small non-enhancement spaces and show the integrity of the gallbladder wall [27].

**Fig. 10:**
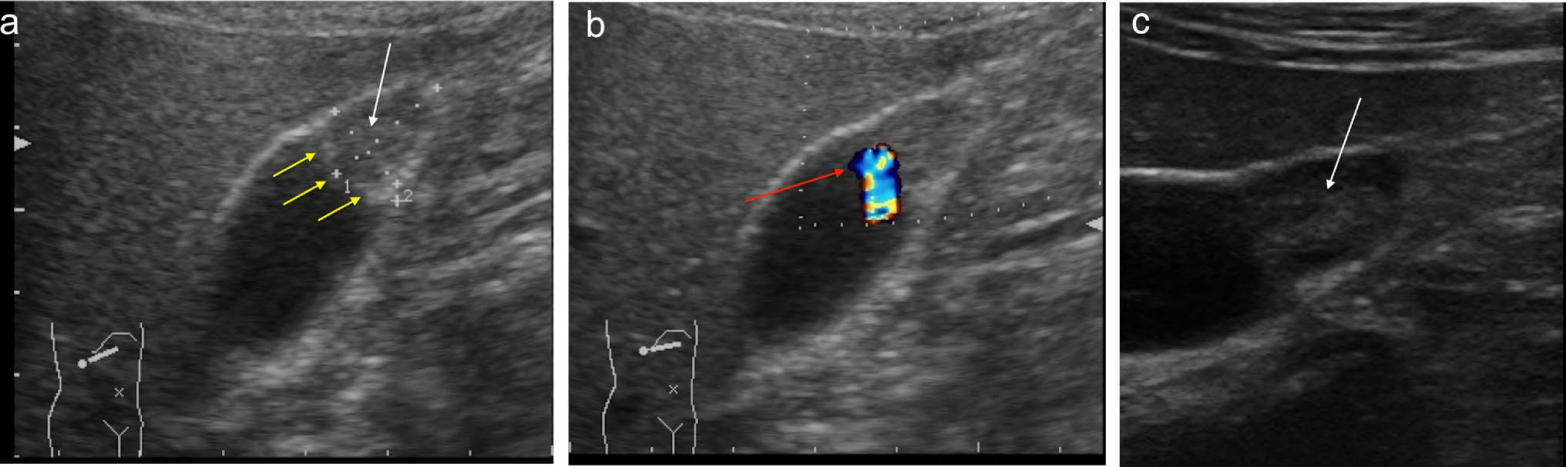
**a** Ultrasound image shows a segmental type of GA that involves a larger portion of the gallbladder wall, the bottom, and the distal third of the body. The affected part appears hypoechoic (white arrows), with hyperechoic cholesterol crystals (yellow arrows). **b** The hyperechoic cholesterol crystal of the segmental type of GA generating twinkling artifacts on the colour Doppler exam (red arrow). **c** The segmental type of GA visualised with a multifrequency linear probe (white arrow)

The segmental type of GA is characterised by the involvement of a larger portion of the gallbladder wall, typically the fundus and the distal third of the body. During sonography the involved portion appears hypoanechoic, often with hyperechoic cholesterol crystals or calcifications generating comet-tail reverberation artifacts, acoustic shadowing, or twinkling artifacts. The uninvolved portion of the GA maintains its normal shape (Fig. 10a–c).

**Fig. 11:**
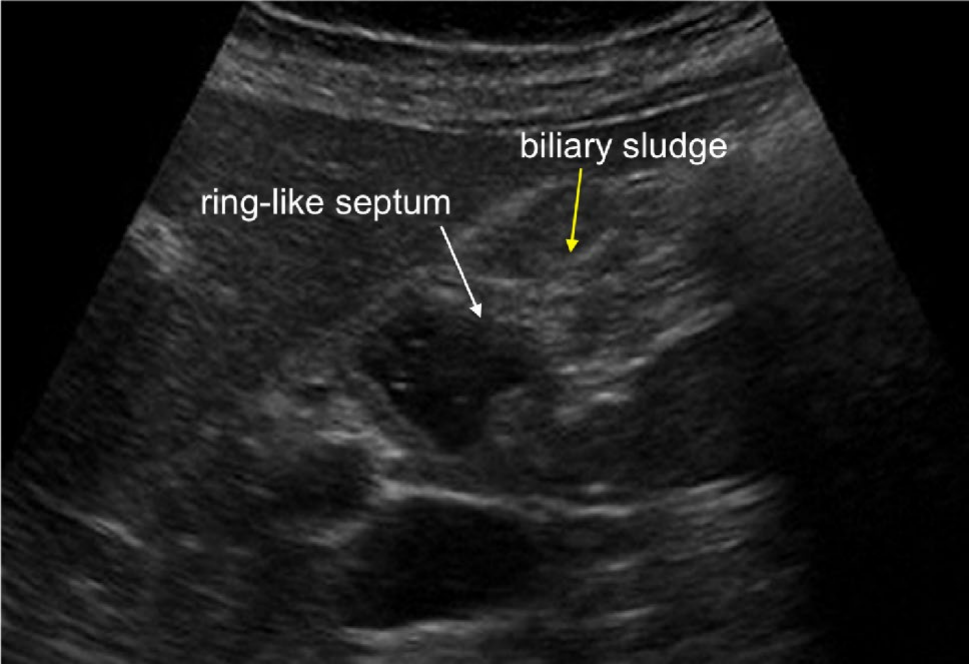
Sonography image depicts annular GA characterised by a ring-shaped (white arrow) circumferential thickening of the gallbladder wall, which usually involves the central part that divides the lumen of the gallbladder into two separate compartments and biliary sludge in the affected area (yellow arrow)

The annular type should be considered a subtype of segmental GA. Annular GA is characterised by a ring-shaped circumferential thickening of the gallbladder wall, which usually involves the central part that divides the lumen of the gallbladder into two separate compartments. During sonography the circumferential ring-like septum can be observed, generally with biliary sludge and stones in the affected area (Fig. 11).

**Fig. 12:**
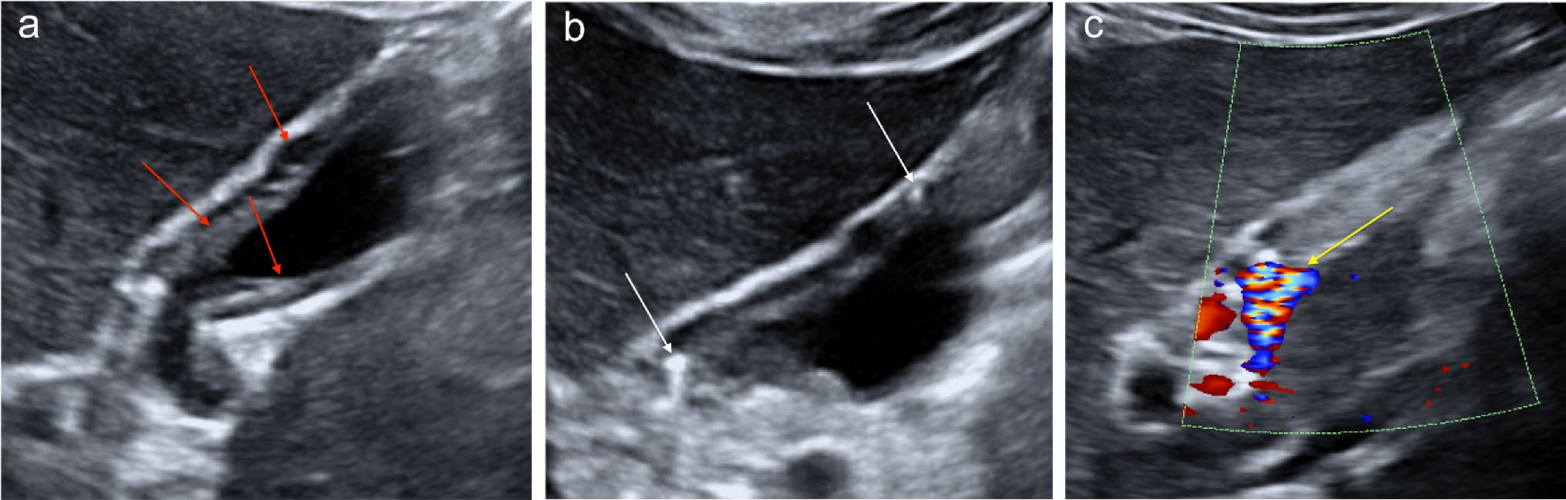
**a** The sonographic images of the diffuse adenomyomatosis type show thickening and irregularity with cholesterol crystals or calcifications along the whole gallbladder wall generating comet-tail reverberation artifacts (white arrows). **b** The sonographic images of the diffuse adenomyomatosis type show thickening and irregularity with cholesterol crystals or calcification along the whole gallbladder wall generating twinkling artifacts on the colour Doppler exam (yellow arrow). **c** The sonographic images of the diffuse adenomyomatosis type show thickening and irregularity (red arrows)

The diffuse adenomyomatosis type is characterised by thickening and irregularity with cholesterol crystals or calcifications along the whole gallbladder wall (Fig. 12a–c).

CEUS is indicated only in the case of differential diagnosis. The main findings include an intact and heterogeneously enhanced gallbladder wall, with some small nonenhanced areas (represented as RAS) in both the arterial phase and the venous phase. The surrounding tissue is normal, without signs of invasion [28].

### True gallbladder polyps: adenoma, carcinoma and metastases

**Fig. 13:**
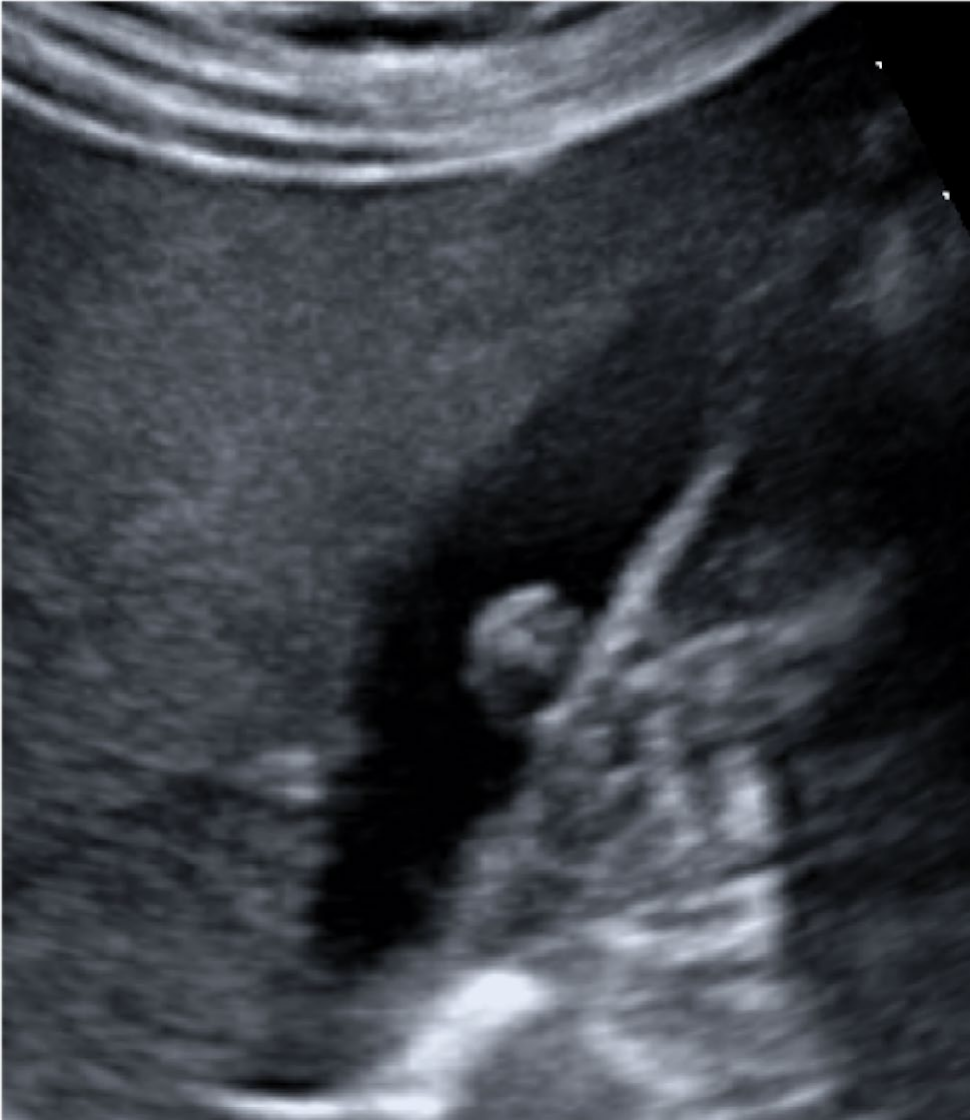
The ultrasound image of the adenoma of the gallbladder appears as a singular homogeneous polyp, isoechoic with the liver parenchyma and sessile

Gallbladder adenoma (also called adenomatous polyp) is a true polyp that can exhibit premalignant behaviour. The frequency of adenomas progressing to adenocarcinoma is unclear [2, 3, 30, 31]. Gallbladder adenoma accounts for 10% of ultrasonographically diagnosed gallbladder polyps. It is habitually solitary with sizes ranging from 5 to 20 mm and can be sessile or pedunculate [29–31]. Gallbladder adenomas have four histological types: pyloric, intestinal, foveolar, and biliary. The main growth patterns of gallbladder adenomas resemble those in colonic adenomas: tubular, papillary (villous), and tubulopapillary (tubulovillous). The tubular type is the most prevalent and is composed of pyloric or intestinal-type glands, but it may show a wide range of morphologic patterns that further complicate the histologic interpretation. Gallbladder adenoma is usually asymptomatic. It may be symptomatic due to cystic duct obstruction by a large adenoma or as a result of associated symptomatic gallstones [29–31]. During sonography, gallbladder adenomas are generally singular homogeneous polyps, often isoechoic with liver parenchyma, sessile or pedunculated (Fig. 13a), and an intralesional vascular spot may be present at the colour Doppler investigation. On CEUS, they usually are homogeneously hyperenhanced in the arterial phase and isoenhanced in the venous phase. The gallbladder wall appears intact and the surrounding tissue normal, with no invasion [28].

**Fig. 14:**
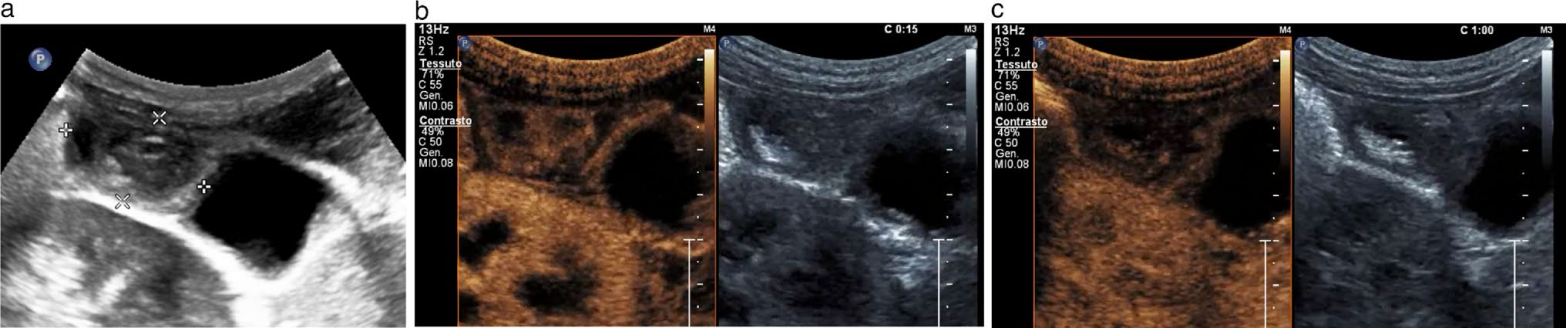
**a** Parietal fundus lesion projecting towards the lumen with a heterogeneous echostructure. **b** The image shows a hyper-enhanced lesion in the arterial phase. **c** The image shows hypo-enhancing in the venous phase

Gallbladder carcinoma is the most common neoplasia of the biliary system and represents approximately 2–4% of malignant tumours. It has a high mortality rate because early diagnosis is rare and generally occurs during advanced stages of the disease because of the scarcity of symptoms. Its diagnosis may be incidental during an abdominal ultrasound. Adenocarcinoma is the most frequent malignant histological type, representing approximately 90% of cases. It is often associated with cholelithiasis (86%), probably due to chronic irritation and inflammation of the gallbladder wall [6, 31, 32]. Three gallbladder carcinoma patterns are described: polyp, mass and thickness pattern. The polyp pattern is the least common, occurring in 15–25% of cases. At sonography, it presents as a polypoid parietal lesion projecting towards the lumen with either a homogeneous or heterogeneous echostructure (Fig. 14a). The most common presentation form of carcinoma is that of a solid mass (approximately 45–65% of cases). During sonography, gallbladder carcinoma appears as a non-homogeneous mass with irregular edges replacing the gallbladder. The mass generally occupies and obscures the gallbladder bed. The wall thickening pattern represents approximately 20–30% of cases. During sonography, this pattern can be found as a focal or diffuse asymmetric wall thickening, usually > 10 mm in size and heterogeneous and irregular [6]. The echo colour Doppler exam generally provides little assistance. Adenoma is unlikely to stand out from gallbladder cancer on echo colour power Doppler examination because both are associated with internal vascularisation [33, 34, 34]. CEUS usually shows a hyper-enhanced lesion in the arterial phase, becoming hypo-enhancing in the venous phase (wash-out) (Fig. 14b, c); the loss of gallbladder wall integrity and the infiltration of surrounding liver tissue are highly suggestive features of malignancy [28].

A study by Kim et al. tested the diagnostic accuracy of HRUS in comparison with conventional transabdominal sonography in the diagnosis of gallbladder neoplasm. The diagnostic accuracy for the T category (TNM staging) by using HRUS was T1a = 92–95%, T1b = 89–95%, T2 = 78–86%, and T3 = 84–89%. The diagnostic accuracy for differentiating T1 from T2 or greater than T2 was 92% and 89% on HRUS and 65% and 70% with conventional transabdominal sonography. Ultrasound features of neoplastic polyps included size greater than 1 cm, single lobular surface, vascular core, hypoechoic polyp and hypoechoic foci. Moreover, HRUS was more accurate in identifying hypoechoic foci in neoplastic polyps, which are strong predictive factors for neoplastic gallbladder polyps [9].

Jang et al. compared HRUS, endoscopic ultrasound (EUS), and computed tomography (CT) in diagnosing and staging gallbladder polyps in 144 patients with gallbladder polyps greater than 10 mm in size. Diagnostic accuracy for malignancy was highest in HRUS compared to other modalities and specificity was the same when using EUS and HRUS [34].

Zhang et al. argued that CEUS had a high accuracy for gallbladder sludge and can help in differential diagnosis among gallbladder polyps, adenoma, and cancer; indeed, in this series of 105 histologically evaluated gallbladder lesions, CEUS showed sensitivity, specificity, and accuracy of 94.1%, 95.5, and 95.2%, respectively, in the differential diagnosis between benign and malignant lesions, as all the sludge masses were unenhanced throughout the study, polyps and adenomas were mostly homogeneously hyperenhanced in the arterial phase and isoenhanced the venous phase, and tumours usually appeared heterogeneously hyperenhanced in the arterial phase and washed out quickly in the venous phase [35]. In particular, CEUS was useful for improving diagnostic accuracy in polyps greater but not less than 10 mm in size [13].

**Fig. 15:**
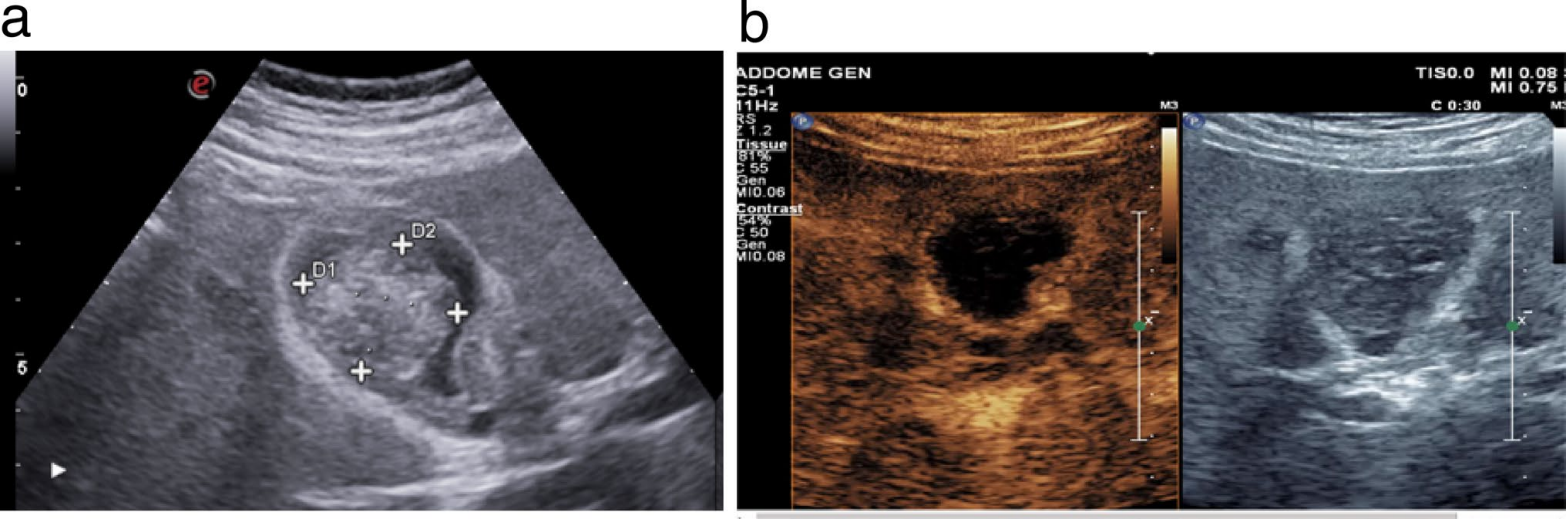
**a** The ultrasound image shows tumefactive biliary sludge that may simulate a gallbladder wall lesion. **b** The CEUS shows the absence of the enhancement of the tumefactive biliary sludge in the early phase

Gallbladder metastases are rare and usually represent an advanced and end stage of malignancy. Nevertheless, in a few patients the gallbladder may represent the first metastatic site. Malignant melanoma, renal cell carcinoma, gastric cancer and hepatocellular carcinoma account for the most common primary malignancies producing gallbladder metastases [36]. From a retrospective analysis carried out by Barella et al. gallbladder metastases appeared as single or multiple polypoid lesions protruding into the gallbladder lumen. Generally, gallbladder polyp metastases have a broad base. Their echogenicity is not intense (lower than usually seen in cholesterol and hyperplastic polyps). In some cases, both biliary sludge and small stones are found. A slight mural thickening can be detected in combination with the luminal vegetations. Colour Doppler analysis is aspecific; in fact, in some cases we can have non-signal detectable or, in other cases, a single central vascular pedicle or multiple spot-like or band-like flow signals are present. When campinable the spectral analysis can reveal an arterial, relatively low-resistance flow, which is a non-specific finding. CEUS can demonstrate lesion vascularisation. Generally, gallbladder metastasis presents moderate to intense contrast enhancement in the arterial phase and a more or less rapid wash-out in the venous phase. In some cases a thin, branching pedicle is recognisable at the centre of the enhanced lesion at real-time CEUS; this enhancing pedicle can be perceptible in the first seconds of microbubble arrival into the lesion or even later [37]. Sometimes the sludge can mimic a gallbladder parietal lesion (Fig. 15a), because does not not move with chang position: this condition is definited as tumefactive biliary sludge [38]. The CEUS shows the absence of the enhancement of the tumefactive biliary sludge in all phases (Fig. 15b), with a sensitivity and specificity of 100% [18].

The differential diagnosis of polyps is summarised in Table 1.

**Table 1:** .

	Type of lesion	Morphology	Echostructure	Color-doppler	Ceus
Pseudopolyps	Cholesterolosis	Parietal foci on the gallbladder wall	Single or multiple hyperechoic parietal foci generating comet-tail artifacts	Twinkling artifacts	Not Indicated
	Cholesterol and Infiammatory Polyps	Pedunculated or sessile and generally smaller than 1 cm	Homogeneous, slightly hyperechoic than liver parenchyma	No intralesional vascularization	Not Indicated
	Gallbladder adenomyomatosis Localized type	Focal thickening usually in the fundus that projects into the lumen in a similar way to a polyp	Hypo-anechoic or with hyperechoic cholesterol crystals or calcifications generating comet-tail reverberation artifacts or acoustic shadowing	Twinkling artifacts	Indicated only in case of differential diagnosis: heterogeneous enhancement, with some small nonenhanced areas (represented as Rokitansky-Aschoff sinuses) on both arterial phase and venous phase. The gallbladder wall intact, surrounding tissue normal, no invasion
True polyps	Gallbladder Adenoma	Singular homogeneous polyps, sessile or pedunculated, sizes ranging from 5 to 20 mm	-Homogeneous or heterogeneous echostructure -Often isoechoic with the liver parenchyma	Intralesional vascular spots may be present	Homogeneously hyperenhanced on arterial phase and isoenhanced on venous phase. The gallbladder wall is intact, surrounding tissue is normal, without invasion
	Gallbladder Carcinoma	Polyp Mass Thickness pattern	-Homogeneous or heterogeneous echostructure -Gallbladder infiltration -Focal or diffuse irregular and asymmetric wall thickening usually > 10 mm in size	Intralesional vascular spots may be present	Hyper-enhancement during the arterial phase with wash-out in venous phase and often demonstrating the infiltration to the adjacent liver
	Gallbladder Metastases	Single or multiple broad base polypoid lesions protruding into the gallbladder lumen	-Echogenicity lower than that of cholesterol and hyperplastic polyps—Mural thickening	Intralesional vascular spots may be present	An intense contrast enhancement in the arterial phase with a thin, branching pedicle at the center of the enhanced lesion can be appreciated. A certain degree of wash-out in the venous phase is usually shown
Pittfall	Biliary Sludge	May simulate pseudopolyps or true polyps	Hyperechoic, moving with changes in the patient’s position, without shadowing unless associated with gallstones	No intralesional vascularization	Indicated only in case of differential diagnosis: not evidence of enhancement and surrounding tissue normal

### Gallbladder management

The goal of sonography is to recognise pseudopolyps and identify true polyps that are potentially malignant. Recently, the joint guidelines released by the European Society of Gastrointestinal and Abdominal Radiology (ESGAR), the European Association for Endoscopic Surgery and other Interventional Techniques (EAES), the International Society of Digestive Surgery—European Federation (EFISDS), and the European Society of Gastrointestinal Endoscopy (ESGE) (2017) have stated that if gallbladder polyps are hyperechoic, multiple, pedunculated, less than 1 cm in size, and without signs of growth on sonography, they may be suggestive of benign pseudopolyps [2].

Generally, the ultrasonographic finding known as comet-tail artifact (or twinkling artifact on colour Doppler exams) in patients with thickened gallbladder lesions is associated with the presence of benign gallbladder diseases, such as cholesterolosis and typical adenomyomatosis, and can be considered a reliable sign of benign gallbladder disease [15]. There is evidence suggesting that pseudopolyps, such as cholesterolosis-related polyps and inflammatory polyps, tend to be smaller than true polyps [2, 3]. If the gallbladder polyp is greater than or equal to 1 cm, cholecystectomy is recommended [1, 3, 25, 26].

If the polyps are less than 1 cm in size, the following risk factors must be considered: age > 50, history of primary sclerosing cholangitis (PSC), Indian ethnicity, and sessile polyps. Gallstones and gallbladder wall thickening are risk factors, but their significance has not yet been established. If the gallbladder polyp is 6–9 mm in a patient with risk factors, cholecystectomy is recommended [2].

If the gallbladder polyp is 6–9 mm in a patient without risk factors, an ultrasound follow-up at 6 months, 1 year and then yearly up to 5 years is recommended. The same behaviour is advocated if the polyp is 5 mm or less but the patient has risk factors. If the gallbladder polyp increases 2 mm or more in size during the ultrasound follow-up, it may be suggestive of malignancy and cholecystectomy is advised [2, 3, 26–28].

## Conclusion

Gallbladder polyps are commonly encountered in daily abdominal sonography practice. Knowledge of the patient’s medical history, as well as the common symptoms and signs, is the mainstay for a correct diagnostic approach. Some ultrasound features of gallbladder polyps can quickly lead to an unequivocal benign or potentially malignant diagnosis. Sonography remains the first choice imaging method for assessing gallbladder polyps and represents an indispensable tool for ensuring appropriate management. It reduces the need for second-level investigations and avoids unnecessary cholecystectomies.
